# 

**Published:** 1897-10

**Authors:** 


### CONTENTS.

ORIGINAL COMMUNICATIONS—

THREATENED DEATH DURING MAJOR ANESTHESIA, WITH A BRIEF DIGRESSION UPON SHOCK. By Robert H. M. Dawbarn, M.D., New York ..... 505
GUNSHOT WOUND OF KIDNEY. OPERATION. RECOVERY. By W. H. Hudson, M.D., LaFayette, Ala ..... 515
FORCEPS-ROTATION IN OCCIPITO-POSTERIOR POSITIONS OF THE VERTEX. By William Gillespie, M.D., Cincinnati, Ohio ..... 519
SUPRAPUBIC CYSTOTOMY FOR STONE. SOME COMPLICATIONS. By Virginius W. Harrison, A.M., M.D., Richmond, Va ..... 525

CORRESPONDENCE—

THE MEDICAL EXPERT—WHAT HE IS AND WHAT HE SHOULD BE ..... 529
ANTITOXIN IN MEMBRANOUS CROUP ..... 532
VENOMOUS SNAKES—A REQUEST ..... 534
OVARIAN CYSTS IN NEGRESSES ..... 536

EDITORIAL—

YELLOW FEVER AND ITS BACILLUS ..... 537
DR. CRAWFORD W. LONG AND ANESTHESIA ..... 540
TRI-STATE MEDICAL SOCIETY OF ALABAMA, GEORGIA AND TENNESSEE ..... 541
NOTICE TO SUBSCRIBERS ..... 543

SELECTIONS AND ABSTRACTS ..... 544
MEDICAL ITEMS ..... 570
BOOK REVIEWS ..... 575
MEDICAL ITEMS—CONTINUED ..... x, xii, xiv, xvi
ADVERTISERS' INDEX TO ADVERTISEMENTS ..... xviii
CLASSIFIED INDEX TO ADVERTISEMENTS ..... xxxvii

